# Depressive Symptoms in Adolescence: Longitudinal Links with Maternal Empathy and Psychological Control

**DOI:** 10.1007/s10802-015-0106-8

**Published:** 2015-12-02

**Authors:** Lente L. A. A. Werner, Jolien Van der Graaff, Wim H. J. Meeus, Susan J. T. Branje

**Affiliations:** Research Centre Adolescence Development, Utrecht University, PO Box 80.140, 3508TC Utrecht, the Netherlands

**Keywords:** Adolescence, Depressive symptoms, Psychological control, Maternal empathy, Self-determination theory

## Abstract

Building on self-determination theory (Deci and Ryan in *Psychological Inquiry*, *11*, 227-268. doi:10.1207/S15327965PLI1104_01, 2000), the aim of the current study was to examine the role of maternal affective and cognitive empathy in predicting adolescents’ depressive symptoms, through mothers’ psychological control use. Less empathic mothers may be less sensitive to adolescents’ need for psychological autonomy, and thus prone to violating this need using psychological control, which may in turn predict adolescents’ depressive symptoms. Moreover, according to interpersonal theory of depression (Coyne in *Journal of Abnormal Psychology*, *85*, 186–193. doi:10.1037/0021-843x.85.2.186, 1976), adolescents’ depressive symptoms may elicit rejecting responses, such as mothers’ psychological control. For six waves, 497 adolescents (57 % boys, *M*_age_ T_1_ = 13.03) annually completed questionnaires on depressive symptoms and maternal psychological control, while mothers reported on their empathy. Cross-lagged path analyses showed that throughout adolescence, both mothers’ affective and cognitive empathy indirectly predicted boys’ and girls’ depressive symptoms, through psychological control. Additionally, depressive symptoms predicted psychological control for boys, and early adolescent girls. These results highlight the importance of (1) mothers’ affective and cognitive empathy in predicting adolescents’ depressive symptoms, and (2) taking gender into account when examining adolescent-effects.

In adolescence, the prevalence of depression increases in boys and girls (e.g., Lewinsohn et al. 1998; Oliva et al. 2014). Because of negative consequences associated with depression (Lewinsohn et al. 1998). and a high chance of recurrence (Hankin 2006). it is important to examine factors that may predict depressive symptoms during adolescence. The development of psychological autonomy in adolescence is thought to be crucial for one’s wellbeing (Deci and Ryan 2000; Wray-Lake et al. 2010). Therefore, violation of the development of psychological autonomy through mothers’ use of psychological control might predict the development of adolescents’ depressive symptoms (Barber 1996). Moreover, mothers who show little empathy might be less responsive to the psychological needs of their adolescent children (Davis 1983). which could make them prone to using psychological control during adolescence. Thus, mothers’ empathy may indirectly predict adolescents’ depressive symptoms, through mothers’ use of psychological control. Additionally, adolescents’ depressive symptoms may evoke negative reactions from parents, and thus also predict mothers’ psychological control use. The aim of this study is to examine relations between maternal empathy, maternal psychological control and boys’ and girls’ depressive symptoms over the course of adolescence.

## Adolescents’ Depressive Symptoms and Maternal Psychological Control

According to self-determination theory (Deci and Ryan 2000). psychological wellbeing is dependent on the satisfaction of three basic psychological needs: feelings of autonomy, competence and relatedness. These needs are essential for ongoing psychological growth, and satisfaction of these needs is associated with most effective functioning. Moreover, as the need for psychological autonomy increases over the course of adolescence (Lansford et al. 2013; Wray-Lake et al. 2010), satisfaction of this need may be particularly important for wellbeing during adolescence. More specifically, if adolescents experience a violation of their need for increasing autonomy, they may turn inward or withdraw, as they learn that expression of psychological autonomy is not accepted (Barber et al. 1994). Indeed, violation of adolescents’ need for autonomy has been found to be an important factor in predicting depressive symptoms in adolescence (e.g., Manzi et al. 2012; Van der Giessen et al. 2014).

A factor which may violate adolescents’ psychological need for autonomy, is mothers’ use of psychological control. Maternal psychological control constrains, invalidates and manipulates adolescents’ psychological and emotional experience and expression, and interferes with their need for psychological autonomy and individuation (Barber 1996; Barber et al. 1994). Experiencing psychological control can thus be considered a restriction of adolescents’ need for autonomy and an intrusion of mothers into adolescents’ establishment of autonomy (Lansford et al. 2013). Across adolescence, normative development of the mother-adolescent relationship is characterized by a peak in conflict intensity around middle adolescence, after which a more egalitarian relationship is established, with more autonomy for the adolescent (De Goede et al. 2009). However, if mothers are psychologically controlling as opposed to granting adolescents autonomy in their relationship, this could predict the development of depressive symptoms. Indeed, more psychological control by mothers has been found to be related to more depressive symptoms in adolescents, both cross-sectionally (e.g., Barber et al. 1994; Oliva et al. 2014) and over time (Barber 1996; Lansford et al. 2013). Adolescents whose mothers used more psychological control in early and middle adolescence showed more depressive symptoms, and more internalizing problems in general compared to adolescents whose mothers used less psychological control (e.g., Barber 1996; Oliva et al. 2014). Thus, maternal psychological control appears to predict adolescents’ depressive symptoms across a large period in adolescence. Moreover, as an increase in conflict intensity between mothers and adolescents is present from early to middle adolescence (De Goede et al. 2009). this may provide an environment in which mothers use psychological control as a reaction to adolescents’ beginning need for autonomy. Although conflict intensity decreases again after middle adolescence, the need for autonomy becomes increasingly salient throughout adolescence (Wray-Lake et al. 2010), which could continue to drive mothers to use psychological control after middle adolescence. Therefore, in this study it is examined whether mothers’ psychological control predicts adolescents’ depressive symptoms throughout adolescence.

## Maternal Psychological Control and Maternal Empathy

Given the expectation that maternal psychological control predicts adolescents’ depressive symptoms throughout adolescence, it is important to examine factors that may precede mothers’ use of psychological control. Empathy has been found to be an important parent-characteristic in determining both positive and negative parenting behaviors (e.g., Gondoli and Silverberg 1997; Perez-Albeniz and De Paul 2003). Empathy is viewed as a multidimensional construct comprising different components. The affective component, also referred to as empathic concern, is an emotional reaction of concern for and compassion with the emotional situation of the other. The cognitive component, also referred to as perspective taking, focuses on the ability to cognitively understand another person’s perspective (Davis 1983). Although it remains understudied, it is widely assumed that both empathic concern and perspective taking play an important role in positive parenting behaviors, as both aspects increase selflessness concern and sensitivity to others (e.g., Davis 1983; Dix 1991; Kochanska 1997). Mothers high in empathic concern may have the capacity to attune to and to be responsive to adolescents’ feelings and may therefore use supportive parenting styles (Dix 1991). Moreover, mothers who have a higher tendency to orient to others’ perspectives may be better aware of adolescents’ needs (Kochanska 1997). such as their need for autonomy. This is supported by the finding that mothers high in perspective taking promoted adolescents’ psychological autonomy more (Gondoli and Silverberg 1997). Thus, both maternal empathic concern and perspective taking are likely to facilitate sensitive responses appropriate to the child’s cues (Davis 1983; Kochanska et al. 2004). and problems in these processes may explain why some mothers do not adequately respond to adolescents’ changing autonomy needs, but instead use psychological control.

Although there is a gap in previous research examining the association between empathy and psychological control, empathy has been found to be related to negative parenting behaviors, with mothers with less empathy being at risk for physical and emotional child abuse (e.g., Rodriguez 2013; Wiehe 2003) which in turn predicted negative child outcomes (Trentacosta and Shaw 2008). Yet, the impact of empathic concern on the one hand, and perspective taking on the other hand remains unclear. Regarding empathic concern, mixed results were found, with mothers’ empathic concern predicting an increased risk for physical abuse in one study (Perez-Albeniz and De Paul 2003), but playing no role in mothers’ risk for physical abuse or neglect in another study (De Paul et al. 2008). Concerning perspective taking, previous findings are also inconsistent, with mothers’ perspective taking either discriminating if mothers’ were at risk for physical abuse (De Paul et al. 2008) or having no effect (Perez-Albeniz and De Paul 2003; Rodriguez 2013). Thus, although both maternal empathic concern and perspective taking can be expected to be negatively related to mothers’ psychological control, there is a lack of research examining this, and results on relations of empathic concern and perspective taking with other aspects of parenting are mixed. Therefore, this study will explore both mothers’ empathic concern and perspective taking tendencies separately in relation to mothers’ psychological control.

## Adolescent-Effects

In addition to the expectation based on self-determination theory (Deci and Ryan 2000) that violation of adolescents’ need for autonomy through mothers’ psychological control predicts adolescents’ depressive symptoms, effects of depressive symptoms on psychological control may also be present. According to Coyne’s interpersonal theory of depression (1976; Branje et al. 2010; Hale 2001). adolescents’ depressive symptoms may evoke negative and rejecting behaviors from parents. In line with this, adolescents’ depressive symptoms might also predict mothers’ psychological control use. When combining self-determination theory (Deci and Ryan 2000) and interpersonal theory of depression (Coyne 1976). these two processes may then create a negative feedback loop of mothers’ psychological control and adolescents’ depressive symptoms, making this relation bidirectional across adolescence. Indeed, previous research in young adolescents found adolescent-effects of depression on psychological control (Barber 1996). Also, higher anxiety levels of adolescents were found to predict higher levels of parental psychological control over the course of adolescence for boys, and for girls from early to middle adolescence (Wijsbroek et al. 2011). Additionally, depressive symptoms were found to predict lower parent-adolescent relationship quality across adolescence (Branje et al. 2010). and more perceived parental rejection (Hale et al. 2008). Although these findings underscore the importance of taking adolescent-effects into account, effects of adolescents’ depressive symptoms on mothers’ psychological control have not yet been examined across adolescence for boys and girls. Therefore, in the present study it is examined if adolescents’ depressive symptoms predict mothers’ psychological control throughout adolescence.

## Gender Differences

Furthermore, gender differences in all associations will be examined. Based on interpersonal theory of depression (Coyne 1976). both boys’ and girls’ depressive symptoms are expected to predict mothers’ psychological control. However, previous research found similar associations to be more consistent across adolescence for boys than for girls (Wijsbroek et al. 2011). In addition, concerning parent-effects, theoretical accounts are diverging. On the one hand, girls may develop more depressive symptoms as a reaction to maternal psychological control, as they have been found to be more susceptible to develop internalizing problems compared to boys (Oliva et al. 2014). On the other hand, since mothers have been found to grant their sons more autonomy (Bumpus et al. 2001). boys may expect more autonomy and thus perceive their mothers’ psychological control as more controlling than girls. In line with both contradicting ideas, previous research found inconsistent results regarding gender differences in the predictive effect of mothers’ psychological control on depressive symptoms during adolescence (e.g., Barber 1996; Lansford et al. 2013). Therefore, in this study, gender differences were explored in the relations between adolescent depressive symptoms, maternal psychological control and maternal empathy across adolescence.

## The Current Study

Given the expected link between adolescents’ depressive symptoms and maternal psychological control, and between maternal psychological control and maternal empathy, the aim of this study was to examine across adolescence if adolescents’ depressive symptoms both predict, and are predicted by mothers’ psychological control, and whether mothers’ psychological control is in turn predicted by mothers’ empathic concern and perspective taking tendencies. This was done by examining (1) the indirect relation over time between maternal empathic concern and perspective taking, and adolescents’ depressive symptoms, via maternal psychological control, through examination of (2) the relation between maternal psychological control and adolescents’ depressive symptoms over time, and (3) the relation between maternal empathic concern and perspective taking and maternal psychological control over time. Moreover, (4) effects from adolescents’ depressive symptoms on maternal psychological control were examined. Finally, (5) gender differences were explored in the relations between maternal empathy, maternal psychological control and adolescents’ depressive symptoms over time. It was expected that throughout adolescence, maternal empathic concern and perspective taking expresses itself in mothers’ use of psychological control, and is thus indirectly related to adolescents’ depressive symptoms. Additionally, no direct link between maternal empathic concern and perspective taking and adolescents’ depressive symptoms was expected, as it was hypothesized that empathy is only important for adolescents’ outcomes when expressed in concrete parenting behaviors, such as psychological control. Moreover, adolescent-effects are expected to be present throughout adolescence. Finally, given contradicting theoretical options, gender differenced were explored.

By studying these questions, this study is the first to not only examine the bidirectional relation between mothers’ psychological control and adolescents’ depressive symptoms, but also to examine the role of mothers’ empathic concern and perspective taking as precursors of mothers’ psychological control use throughout adolescence.

## Methods

### Participants

The current study used six waves of data from the ongoing longitudinal study Research on Adolescent Development and Relationships-Young (RADAR). Data from 497 adolescents (57 % boys) and their mothers was used, who were followed annually for 6 years. At the first wave, adolescents attended the first year of secondary education, and were on average 13.03 years (*SD* = 0.46, range: 11.01–15.56 years).Their mothers were on average 44.41 years (*SD* = 4.45, range: 30.83–64.16 years). Moreover, 95 % of the adolescents was of Dutch ethnicity, and 88 % came from a family with medium or high socioeconomic status (SES; at least one parent holding a medium or high level job). Of the original sample, 12.5 % (62 participants) was no longer involved in the study at the sixth wave. These 62 participants were significantly older, *F*(1, 495) = 4.30, *p* = 0.039, and of lower SES families, *F*(1, 487) = 10.30, *p* < 0.001, than participants still participating in the sixth wave.

### Procedure

Participants were recruited in the western and central parts of The Netherlands. All adolescents and their parents received written information about the study. Confidentiality was ensured, after which parents provided active written informed consent to participate. Adolescents and mothers simultaneously, but independent from each other, completed questionnaires during 1-h annual home visits, and trained interviewers provided additional verbal instructions. In each wave, adolescents and mothers each received a monetary reward (approximately €25) for participating. The study was approved by the Medical Ethics Committee of the University Medical Centre Utrecht.

### Measures

#### Depressive Symptoms

Adolescents reported on their depressive symptoms using the Reynolds Adolescent Depression Scale-Second Edition (RADS-2; Reynolds 2000). The RADS-2 assesses negative self-evaluations, dysphoric mood and somatic complaints using 23 items (e.g., “I feel lonely”). Items were assessed on a 4-point Likert-scale ranging from 1 (*Almost never*) to 4 (*Usually*). All scores were summed to calculate a total depressive symptoms score, with higher scores representing more depressive symptoms. Good psychometric properties, such as internal consistency and validity have been established (Osman et al. 2010). Cronbach’s alphas ranged from 0.93 to 0.95 across the six waves.

#### Psychological Control

Adolescents reported on their mothers’ psychological control using the Psychological Control Scale-Youth Self Report (PCS-YSR; Barber 1996). This scale consists of eight items and assesses adolescents’ perceptions of the extent to which mothers invalidate their feelings and expressions, use personal attacks and withdraw love (e.g., “My mother often interrupts me”). All items were answered on a 5-point Likert-scale, ranging from 1 (*Does not describe my mother at all*) to 5 (*Describes my mother very well*). All items were summed to calculate a total psychological control score, with higher scores implying more psychological control. Both reliability and external validity of the PCS-YSR were established (Barber 1996). In this sample, Cronbach’s alphas ranged from 0.82 to 0.90 across the six waves.

#### Empathy

Mothers reported on their empathic tendencies using two scales of the Interpersonal Reactivity Index (IRI; Davis 1983). empathic concern and perspective taking. Empathic concern consists of seven items assessing the tendency to sympathize with others in need (e.g., “I often have tender, concerned feelings for people less fortunate than me”). Perspective taking consists of seven items assessing the tendency to spontaneously adopt the others’ point of view (e.g., “I try to look at everybody’s side of a disagreement before I make a decision”). All items were measured on a 5-point Likert-scale ranging from 0 (*Does not describe me well*) to 4 (*Describes me very well*), with a higher score implying more empathic tendencies. Internal consistency, external validity and construct validity of the IRI were established (Hawk et al. 2012). For perspective taking, Cronbach’s alphas ranged from 0.74 to 0.81 across waves. For empathic concern, Cronbach’s alphas ranged from 0.66 to 0.71.

### Statistical Analyses

To examine the hypothesized 6-year bidirectional associations between adolescents’ depressive symptoms, maternal psychological control and maternal empathy, cross-lagged path analyses were performed in M*plus* Version 6.12 (Muthén and Muthén 1998–2010). Across all measures and waves, on average 10 % of the responses were missing per scale (ranging from 0.4 % to 16.3 %). Little’s Missing Completely At Random test (MCAR; Little 1988) revealed a normed χ^2^ (χ^2^/df) value of 1.19 (859.43/722), indicating a good fit between the sample scores with and without imputation (Bollen 1989). Therefore, missing data could be estimated in M*plus* using the Full Information Maximum Likelihood approach (FIML), allowing information of all 497 participants to be used in all waves (Muthén and Muthén 1998–2010; Widaman 2006).

Series of multigroup cross-lagged path analyses were used to construct the best fitting model separately for empathic concern (EC) and perspective taking (PT). First, for both constructs, a baseline model was constructed that included all 1- and 2-year stability paths of adolescents’ depressive symptoms, maternal psychological control and maternal empathy, all within-wave correlations and all cross-lagged paths. Next, time-invariance (i.e., if a relation is stable over time) was tested for stability paths and within-wave correlations by constraining identical paths to be equal to each other across the six waves separately for boys and girls. As these models are nested, χ^2^-difference tests were computed to examine whether constraining paths significantly worsened model fit. If this was not the case, the relations were constrained to be equal over time. Second, gender differences were tested by constraining stability paths and within-wave correlations to be equal between boys and girls and testing whether this significantly worsened model fit. The resulting models were used as starting point to test change over time in the longitudinal relations between boys’ and girls’ depressive symptoms, maternal psychological control and maternal empathy, by constraining the cross-paths over time per relation. Subsequently, to test gender differences in these relations, cross-paths were constrained across gender. If no change over time was found for a relation, all waves were constrained across gender in one step. If a relation did change over time, these cross-paths were constrained across gender per wave, to examine when in adolescence potential gender differences were present.

These steps resulted in a final EC and PT model, in which longitudinal indirect effects from maternal empathy to adolescents’ depressive symptoms via maternal psychological control and adolescent-effects were tested using the model indirect-command in M*plus*. As both depressive symptoms and psychological control were positively skewed, bootstrapping was used to account for non-normality of both constructs and of the indirect effects (Mackinnon et al. 2004; Preacher and Hayes 2004; Preacher and Hayes 2008). In bootstrapping, random samples are generated based on the original data (1000 sets in this data), leading to a distribution of 1000 estimates of direct and indirect effects, on which 95 % confidence intervals (CI) are based. The fit of the models was evaluated using the Comparative Fit Index (CFI) and the Root Mean Square Error of Approximation (RMSEA). The model fit is considered to be good if the CFI is 0.95 or higher and the RMSEA is 0.06 or below. As the χ^2^-test is influenced by sample size (Hu and Bentler 1999). χ^2^ was only used to compare nested models and not to assess model fit.

## Results

### Descriptive Statistics

Multivariate Analysis of Variance (MANOVA) showed that mean depressive symptoms scores were significantly higher for girls compared to boys at all ages, range: *F*(1, 348) = 13.16, *p* < 0.001–*F*(1, 348) = 50.30, *p* < 0.001 (see Table 1). Also, maternal psychological control scores from age 16 to 18 were significantly higher for girls than boys, range: *F*(1, 348) = 5.28, *p* = 0.022–*F*(1, 348) = 7.47, *p* = 0.015. Moreover, the mean score of perspective taking at age 13, *F*(1, 348) = 3.95, *p* = 0.048, and age 18, *F*(1, 348) = 4.43, *p* = 0.036, was significantly higher for mothers of boys than mothers of girls. Stabilities between successive waves ranged from 0.53 to 0.80 for depressive symptoms, from 0.35 to 0.74 for psychological control, from 0.63 to 0.75 for empathic concern, and from 0.64 to 0.78 for perspective taking. As expected, within-wave bivariate correlations of depressive symptoms and psychological control were consistently significantly positive (range 0.33–0.46), as were the correlations of perspective taking and empathic concern (range 0.50–0.54). Moreover, while empathic concern was not significantly related to depressive symptoms at all (range − 0.06–0.02), perspective taking is significantly negatively related to depressive symptoms at age 16 and 17 (range − 0.15– -0.05). Finally, perspective taking was significantly negatively related to psychological control at age 13, 16, 17 and 18 (range − 0.21– -0.01), while for empathic concern, this was only the case at age 17 (range − 0.10–0.06).^1^

**Table 1:** Mean levels and standard deviations of all studied variables from age 13 to 18 for the total sample (*N* = 497) and boys (*N* = 283) and girls (*N* = 214) separately

Variable	Age 13 *M* (*SD*)	Age 14 *M* (*SD*)	Age 15 *M* (*SD*)	Age 16 *M* (*SD*)	Age 17 *M* (*SD*)	Age 18 *M* (*SD*)
Total sample						
Empathic concern M	20.14 (3.34)	20.79 (3.29)	20.84 (3.47)	20.62 (3.43)	20.85 (3.39)	20.83 (3.47)
Perspective taking M	18.39* (3.55)	19.14 (3.55)	19.40 (3.49)	19.53 (3.74)	19.68 (3.73)	19.62* (3.82)
Psychological control M	14.18 (5.23)	13.97 (5.45)	14.18 (5.73)	14.89* (5.94)	14.39* (5.91)	14.44* (6.12)
Depressive symptoms A	37.55** (11.34)	34.60** (11.43)	35.25** (12.05)	35.90** (12.33)	35.37** (11.78)	36.60** (12.55)
Boys						
Empathic concern M	20.20 (3.38)	20.74 (3.22)	20.96 (3.37)	20.66 (3.47)	20.94 (3.34)	20.78 (3.33)
Perspective taking M	18.64 (3.55)	19.31 (3.37)	19.62 (3.74)	19.92 (3.52)	19.88 (3.71)	19.62 (3.82)
Psychological control M	14.22 (5.31)	13.79 (5.36)	13.71 (5.43)	14.29 (5.64)	13.84 (5.49)	13.91 (6.02)
Depressive symptoms A	35.82 (10.28)	31.89 (9.13)	31.63 (8.23)	32.58 (9.20)	32.41 (8.67)	34.05 (10.80)
Girls						
Empathic concern M	20.07 (3.29)	20.84 (3.39)	20.68 (3.60)	20.58 (3.39)	20.74 (3.45)	20.89 (3.66)
Perspective taking M	18.07 (3.55)	19.91 (3.76)	19.11 (3.63)	19.39 (3.74)	19.37 (3.97)	19.27 (3.94)
Psychological control M	14.13 (5.13)	14.20 (5.56)	14.81 (6.07)	15.67 (6.24)	15.08 (6.35)	15.16 (6.19)
Depressive symptoms A	39.82 (12.25)	38.21 (13.10)	40.08 (14.45)	40.25 (14.41)	39.19 (13.99)	40.05 (13.90)

Empathic concern and perspective taking were measured by summing seven items with a scale from 0 to 4. Psychological control was measured by summing eight items with a scale from 1 to 5. Depressive symptoms were measured by summing 23 items with a scale from 1 to 4

*M* Maternal variables, *A* Adolescent variables

*Significantly different between boys and girls at *p* < 0.05

**Significantly different between boys and girls at *p* < 0.01

### Model Construction

The baseline models including all 1- and 2-year stability paths of all constructs, all within-wave correlations and all cross-lagged paths showed adequate model fit, with CFI = 0.97, RMSEA =0.06, χ^2^(156) = 304.41, *p* < 0.001 for EC, and CFI = 0.97, RMSEA =0.06, χ^2^(156) = 298.88, *p* < 0.001 for PT. The tests for time-invariance showed that stability paths could not be constrained, but changed over time for EC, Δχ^2^(42) = 62, *p* = 0.024, and PT, Δχ^2^(42) = 85.68, *p* < 0.001. Yet, all within-wave correlations could be constrained over time for EC, Δχ^2^(24) = 24.29, *p* = 0.445, and PT, Δχ^2^(24) = 15.40, *p* = 0.909. Subsequently, constraining the within-wave correlations across gender did not significantly worsen model fit for EC, Δχ^2^(27) = 32.82, *p* = 0.203, and PT, Δχ^2^(27) = 38.83, *p* = 0.066. This led to a good model fit for EC, CFI = 0.96, RMSEA =0.06, χ^2^(183) = 343.237, *p* < 0.001, and for PT, CFI = 0.97, RMSEA =0.06, χ^2^(183) = 331.70, *p* < 0.001. These models were used as starting point to examine time-invariance and gender differences in the longitudinal relations between EC and PT, psychological control and depressive symptoms.

#### Longitudinal Relations

When testing change over time in the longitudinal relations, all cross-paths were time-invariant, except for the bidirectional relation between depressive symptoms and psychological control for girls, and the relations from EC and PT to psychological control for boys (see Table 2), indicating that the strength of most relations remained similar throughout adolescence. Next, when examining gender differences in the time-invariant relations from psychological control to EC and PT, and the bidirectional relation between depressive symptoms and EC and PT, constraining these relations across gender did not worsen model fit, range: Δχ^2^(4) = 0.00, *p* = 0.956–Δχ^2^(1) = 3.29, *p* = 0.070, indicating that no gender differences were present. Next, for the relations that did change over time for either boys or girls, gender differences were tested for each wave separately. First, gender differences were tested per wave for the bidirectional relation between psychological control and depressive symptoms, which changed over time for girls. For the relation from psychological control to depressive symptoms, this relation could not be constrained across gender at age 15 to 16 and 17 to 18, range: Δχ^2^(1) = 4.76, *p* = 0.029–Δχ^2^(1) = 5.12, *p* = 0.024, while constraining was possible at all other ages, range: Δχ^2^(1) = 0.13, *p* = 0.718–Δχ^2^(1) = 1.75, *p* = 0.186. When constraining the relation from depressive symptoms to psychological control across gender per wave, for both EC and PT this did not worsen model fit at all ages, indicating that no gender differences were present, range: Δχ^2^(1) = 0.17, *p* = 0.679–Δχ^2^(1) = 3.78, *p* = 0.052. However, this absence of gender differences is contrary to χ^2^-difference tests showing that overall, this relation changed over time for girls and not for boys. Because of capitalization on chance, the tests on gender differences per wave were less reliable than the overall χ^2^-difference tests, making it thus impossible to indicate at which age gender differences were present. Therefore, all waves of this relation were freely estimated for boys and girls. Next, for the relations from EC and PT to psychological control, which changed over time for boys, only at age 13 to 14 for EC, Δχ^2^(1) = 8.88, *p* = 0.003, and at age 14 to 15 for PT, Δχ^2^(1) = 9.22, *p* = 0.002, these relations could not be constrained across gender. At all other ages, no gender differences were present, range: Δχ^2^(1) = 0.01, *p* = 0.924–Δχ^2^(1) = 3.79, *p* = 0.052.

**Table 2:** χ^2^-Difference test results of time-invariance tests of longitudinal relations for boys (*N* = 283) and Girls (*N* = 214)

Baseline model used to test time-invariance against per relation	χ^2^	*df*	*p*	χ^2^	*df*	*P*
	Empathic concern			Perspective taking		
	343.24	183	< 0.001	331.70	183	< 0.001
	Δχ^2^	Δ*df*	*p*	Δχ^2^	Δ*df*	*p*
Boys						
Empathy M → Psychological control M	10.32	4	0.035	9.78	4	0.044
Psychological control M → Empathy M	2.65 ^a^	4	0.619	3.05 ^a^	4	0.349
Psychological control M → Depression A	1.59 ^a^	4	0.811	1.85 ^a^	4	0.764
Depression A → Psychological control M	3.03 ^a^	4	0.553	2.42 ^a^	4	0.660
Depression A → Empathy M	1.81 ^a^	4	0.771	2.80 ^a^	4	0.592
Empathy M → Depression A	3.11 ^a^	4	0.540	7.77 ^a^	4	0.101
Girls						
Empathy M → Psychological control M	2.34 ^a^	4	0.674	4 ^a^	4	0.406
Psychological control M → Empathy M	0.43 ^a^	4	0.980	1.71 ^a^	4	0.789
Psychological control M → Depression A	12.10	4	0.017	12.40	4	0.015
Depression A → Psychological control M	12.37	4	0.015	12.89	4	0.012
Depression A → Empathy M	2.21 ^a^	4	0.697	4.70 ^a^	4	0.320
Empathy M → Depression A	4.43 ^a^	4	0.351	0.79 ^a^	4	0.940

*M* Maternal variables, *A* Adolescent variables

^a^Adding time constraints did not significantly worsen model fit. Therefore, these paths were constrained over time

This resulted in two models with gender differences in the relations from depressive symptoms to psychological control from age 13 to 18, from psychological control to depressive symptoms at age 15 to 16 and 17 to 18, and in the relation from empathy to psychological control at age 13 to 14 for EC, and at age 14 to 15 for PT. These two final models showed a good fit to the data, with CFI = 0.97, RMSEA =0.05, χ^2^(226) = 371.96, *p* < 0.001 for EC, and CFI = 0.97, RMSEA =0.05, χ^2^(229) = 374.80, *p* < 0.001 for PT. In addition, in these final models, medium to large proportions of variance were explained in all variables. That is, for EC, the percentage of variance explained was 29.5 %–64.3 % in adolescents’ depressive symptoms, 14 %–52.7 % in maternal psychological control, and 43.9 %–66.6 % in maternal empathic concern. For PT, this was 29.4 %–64.7 % in adolescents’ depressive symptoms, 12.7 %–56.3 % in maternal psychological control, and 44.9 %–67.4 % in maternal perspective taking.

### Relations Between Adolescents’ Depressive Symptoms, Maternal Psychological Control and Maternal Empathy

**Fig. 1:**
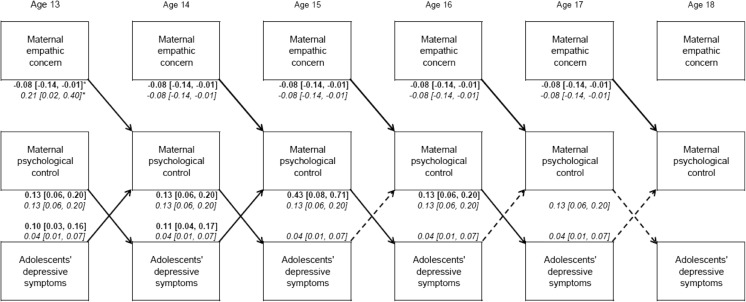
Unstandardized results of the longitudinal relations of EC, psychological control and depressive symptoms significant at *p* < 0.05. For reasons of clarity, non-significant cross-paths, within-wave correlations and stability paths are not depicted. Solid arrows indicate significant paths for boys and girls. Dotted arrows indicate significant paths for boys. Estimates are printed in bold for girls and in italic for boys. 95 % CI is shown between brackets. * Significantly different between boys and girls at *p* < 0.05 when comparing the 95 % CIs

**Fig. 2:**
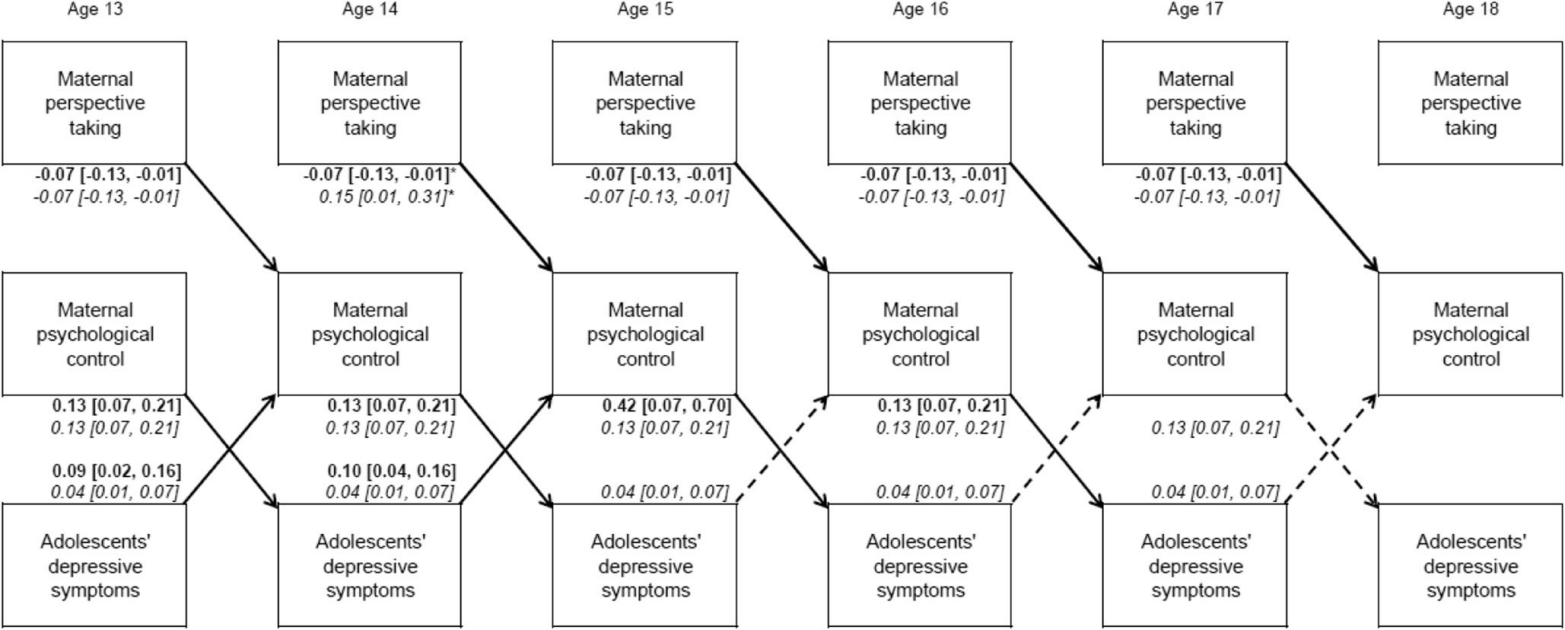
Unstandardized results of the longitudinal relations of PT, psychological control and depressive symptoms significant at *p* < 0.05. For reasons of clarity, non-significant cross-paths, within-wave correlations and stability paths are not depicted. Solid arrows indicate significant paths for boys and girls. Dotted arrows indicate significant paths for boys. Estimates are printed in bold for girls and in italic for boys. 95 % CI is shown between brackets. * Significantly different between boys and girls at *p* < 0.05 when comparing the 95 % CIs

As is shown in Fig. 1 (EC) and Fig. 2 (PT), the relation from maternal psychological control to both aspects of maternal empathy and the bidirectional associations between adolescents’ depressive symptoms and maternal empathy were not significant. Yet, in both models, adolescent- and parent-effects were found. That is, more maternal psychological control predicted more depressive symptoms of adolescents one year later, and more depressive symptoms predicted more psychological control the subsequent year (Table 3). For boys, these bidirectional effects were present at all ages. However, for girls, parent-effects were present until age 16 to 17, and adolescent-effects until age 14 to 15. Moreover, more maternal empathic concern and perspective taking predicted lower maternal psychological control over time for girls. For boys, this relation was also present, but was positive, and significantly different from girls at age 13 to 14 for EC and at age 14 to 15 for PT (see Fig. 1 and Fig. 2).

**Table 3:** Standardized results of all longitudinal relations from age 13 to age 18 for boys (*N* = 283) and girls (*N* = 214)

Longitudinal relation	Empathic concern					Perspective taking				
	*β*_13-14_	*β*_14-15_	*β*_15-16_	*β*_16-17_	*β*_17-18_	*β*_13-14_	*β*_14-15_	*β*_15-16_	*β*_16-17_	*β*_17-18_
Boys										
Empathy M → Psychological control M	0.13*	−0.04*	−0.04*	−0.04*	−0.04*	−0.04*	0.10*	−0.04*	−0.04*	−0.04*
Psychological control M → Empathy M	−0.01	−0.01	−0.01	−0.01	−0.01	−0.03	−0.03	−0.03	−0.03	−0.03
Psychological control M → Depression A	0.07*	0.08*	0.07*	0.08*	0.07*	0.07*	0.08*	0.07*	0.08*	0.07*
Depression A → Psychological control M	0.08*	0.07*	0.06*	0.07*	0.07*	0.08*	0.07*	0.06*	0.07*	0.06*
Depression A → Empathy M	−0.02	−0.02	−0.01	−0.02	−0.02	−0.01	−0.01	−0.01	−0.01	−0.01
Empathy M → Depression A	−0.01	−0.01	−0.01	−0.01	−0.01	0.02	0.02	0.02	0.02	0.02
Girls										
Empathy M → Psychological control M	−0.05*	−0.04*	−0.05*	−0.04*	−0.04*	−0.04*	−0.04*	−0.04*	−0.04*	−0.04*
Psychological control M → Empathy M	−0.04	−0.04	−0.04	−0.04	−0.04	−0.03	−0.03	−0.03	−0.03	−0.03
Psychological control M → Depression A	0.05*	0.05*	0.18*	0.06*	−0.07	0.05*	0.05*	0.18*	0.06*	−0.07
Depression A → Psychological control M	0.22*	0.23*	0.01	0.08	0.01	0.21*	0.23*	0.01	0.07	0.01
Depression A → Empathy M	0.00	0.00	0.00	0.00	0.00	−0.01	−0.01	−0.01	−0.01	−0.01
Empathy M → Depression A	0.02	0.02	0.02	0.02	0.02	0.02	0.02	0.02	0.02	0.02

*M* Maternal variables, *A* Adolescent variables

*Significant at *p* < 0.05

### Psychological Control as a Mediator

Finally, indirect effects were found from both maternal empathic concern and perspective taking to adolescents’ depressive symptoms, via maternal psychological control (see Table 4). These indirect effects were present for boys throughout adolescence, while for girls, they were present until age 17. No direct effect from maternal empathic concern and perspective taking to adolescents’ depressive symptoms, or from depressive symptoms to empathy, were found.

**Table 4:** Indirect effects from age 13 to 18 from maternal empathy to adolescents’ depressive symptoms, via maternal psychological control (*N* = 497)

	Age 13–Age 15			Age 14–Age 16			Age 15–Age 17			Age 16–Age 18		
	*β*	*B*	*95* % *CI*	*β*	*B*	*95* % *CI*	*β*	*B*	*95* % *CI*	*β*	*b*	*95* % *CI*
Empathic concern												
Girls	−0.002	−0.010*	−0.021,-0.002	−0.008	−0.032*	−0.073,-0.003	−0.003	−0.010*	−0.021,-0.002	0.003	0.012	−0.007,0.045
Boys	0.011	0.026*	0.005,0.063	−0.003	−0.010*	−0.021,-0.002	−0.003	−0.010*	−0.021,-0.002	−0.003	−0.010*	−0.021,-0.002
Perspective taking												
Girls	−0.002	−0.009*	−0.021,-0.002	−0.007	−0.028*	−0.073,-0.003	−0.002	−0.009*	−0.021,-0.002	0.003	0.011	−0.004,0.042
Boys	−0.004	−0.009*	−0.021,-0.002	0.007	0.019*	0.003,0.046	−0.003	−0.009*	−0.021,-0.002	−0.003	−0.009*	−0.021,-0.002

*Significant at *p* < 0.05

## Discussion

Since the prevalence of depression increases when children enter adolescence (Lewinsohn et al. 1998; Oliva et al. 2014). the aim of this study was to extend our understanding of parental factors predicting adolescents’ depressive symptoms. Additionally, this study also aimed at understanding the opposite effect of adolescents’ depressive symptoms predicting parenting practices. Both maternal empathic concern and perspective taking predicted mothers’ use of psychological control, which in turn predicted adolescents’ depressive symptoms. These findings show that, although mothers’ empathy was not directly related to adolescents’ depressive symptoms, both aspects of mothers’ empathy are important in predicting adolescents’ depressive symptoms, through the use of psychological control. Moreover, these findings are consistent with self-determination theory stating that violation of psychological autonomy in adolescence may result in decreased wellbeing (Deci and Ryan 2000). Additionally, adolescents’ depressive symptoms predicted mothers’ psychological control, thus supporting interpersonal theory of depression that adolescents’ depressive symptoms may elicit rejecting reactions of parents (Coyne 1976).

From the perspective of self-determination theory (Deci and Ryan 2000). the finding that more psychological control by mothers predicted more depressive symptoms of adolescents implies that violation of adolescents’ need for psychological autonomy is indeed related to lower wellbeing. More specifically, adolescents who experience psychological control may thus turn inward and develop depressive symptoms, as they learn that their psychological autonomy is not accepted (Barber et al. 1994). For boys, this relation was found consistently from age 13 to 18. Yet, girls’ depressive symptoms were predicted by maternal psychological control only until age 17, while girls remained to experience higher levels of depressive symptoms and psychological control than boys at this age. This implies that, although girls remain to experience psychological control and depressive symptoms, mothers’ psychological control is no longer of impact on girls’ depressive symptoms. This may be because generally, girls start their transition towards independence and adult roles at an earlier age compared to boys (Cohen et al. 2003). which may result in girls being no longer affected by their mothers’ psychological control at age 17, while for boys this relation remains to exist.

Moreover, both mothers’ empathic concern and perspective taking tendencies were found to predict mothers’ psychological control use, with higher empathic tendencies predicting lower psychological control. Although previous research showed inconsistent results regarding the importance of empathic concern and perspective taking for parenting (De Paul et al. 2008; Perez-Albeniz and De Paul 2003), both aspects were found to be of similar importance for predicting mothers’ psychological control. Mothers low in empathic concern and perspective taking are thus prone to use psychological control, as they may be less aware of, and emotionally responsive to adolescents’ needs (Davis 1983). such as adolescents’ increasing need for psychological autonomy (Wray-Lake et al. 2010). This negative association was found throughout adolescence for girls for both empathic concern and perspective taking. For boys, it was consistently found between age 15 and 18, but remarkably, this relation was positive between age 13 and 14 for empathic concern, and between age 14 and 15 for perspective taking. This might be because in early adolescence, a temporary decrease of boys’ own empathic concern and perspective taking tendencies (Van der Graaff et al. 2014). could coincide with boys being less open to empathy in general, which may result in a perception of more empathic mothers’ as being more controlling. Another explanation could be that because parents typically grant boys more autonomy than girls (Bumpus et al. 2001). boys are more likely to perceive their mothers as more controlling than girls, even when those mothers show more empathic abilities. Subsequently, when the parent-adolescent relationship becomes increasingly egalitarian towards late adolescence (De Goede et al. 2009). boys may no longer perceive their empathic mothers as controlling, and only less empathic mothers remain to exert more psychological control.

As expected, low empathic concern and perspective taking tendencies of mothers indirectly predicted adolescents’ depressive symptoms, through mothers’ increased psychological control use. This indirect effect was found for boys throughout adolescence, and for girls until middle adolescence. The absence of a direct association between mothers’ empathic concern and perspective taking, and adolescents’ depressive symptoms, suggests that mothers’ empathic tendencies are only important for predicting adolescents’ depressive symptoms when they are expressed in concrete parenting behaviors such as psychological control, which in turn predict adolescents’ depressive symptoms.

In line with previous research supporting bidirectional effects between adolescents’ depressive symptoms and parents’ autonomy support (Van der Giessen et al. 2014). adolescents’ depressive symptoms were also predictive of mothers’ psychological control use. This supports interpersonal theory of depression (Coyne 1976). in that adolescents’ depressive behaviors evoke negative and rejecting reactions from others. However, adolescents with more depressive symptoms may also perceive their mothers as more psychologically controlling than adolescents’ reporting less depressive symptoms, because of negative cognitions and increased perceptions of rejection associated with their depressive symptoms (Hale et al. 2008; Lewinsohn et al. 1998). For boys, adolescent-effects were present throughout adolescence. However, for girls, adolescent-effects were only present in early adolescence. This may be because girls have a higher tendency to direct their depressive feelings inward than boys (Bennett et al. 2005). Girls’ tendency to show self-oriented symptoms such as self-blame, feelings of failure and guilt, rather than more general symptoms such as depressed mood and lack of enjoyment, may have less impact on the relationship with mothers, in particular in middle and late adolescence, when relationship quality increases (De Goede et al. 2009).

## Limitations and Strengths

Despite the six-wave longitudinal design of the study and its multi-informant measures, the current findings should be viewed in light of some limitations. Firstly, as it is important to address adolescents’ perception when studying factors that predict their depressive symptoms, maternal psychological control was reported by adolescents. However, this measure might reflect a tendency of adolescents with more depressive symptoms to interpret their environment, and thus their mothers’ psychological control use, in a more negative way (Lewinsohn et al. 1998). Yet, mothers’ psychological control was found to be consistently predicted by mothers’ self-reported empathy, suggesting that the current assessment of psychological control is a reliable representation of mothers’ psychological control use. Nonetheless, using information of both adolescents and mothers, potentially with additional observational measures, might further clarify the relations examined in this study. Secondly, only mothers as primary caregivers were included in this study. Previous research did not examine the relation between paternal empathy and negative parenting behaviors (e.g. De Paul et al. 2008; McElroy and Rodriguez 2008). thus ignoring the unique role of fathers in parenting and adolescents’ depressive symptoms development (Rueger et al. 2014). Future research should include fathers to examine the role of their empathic tendencies in parenting and adolescents’ depressive symptoms. Furthermore, as a community sample was used in this study, caution is necessary when generalizing the findings to clinically depressed adolescents. As the findings of this study provide a first basis in unraveling importance of mothers’ empathic concern, perspective taking and psychological control for adolescents’ depressive symptoms, it would be interesting to examine if the found relations are similar in adolescents suffering from clinical depression. Also, since the participants in this study were relatively high functioning and predominantly of Dutch ethnicity, generalizability of the findings to families with lower SES and of non-western ethnicity is limited as well. Nonetheless, a cross-cultural study found that the relation between psychological control and adolescents’ depressive symptoms is similar between Belgian and South-Korean adolescents (Soenens et al. 2012). suggesting that the current findings may be at least partially generalizable across ethnicity. However, future research should use a more diverse sample in terms of SES and ethnicity. Finally, one-year intervals were used to examine change in adolescence as a period of relative stability. However, it would be interesting to examine these relations using smaller intervals, to better grasp the daily bidirectional interactions between mothers and adolescents that underlie the found processes.

Notwithstanding these limitations, the current study increases our understanding of factors important for predicting depressive symptoms in adolescence. Despite the lack of direct associations between mothers’ empathy and adolescents’ depressive symptoms, this study is the first to show over a 6-year period that both aspects of mothers’ empathy are indeed important in predicting boys’ and girls’ depressive symptoms in adolescence, with mothers’ use of psychological control as the underlying mechanism. Moreover, the findings support self-determination theory (Deci and Ryan 2000). by underscoring the importance of specific parenting behaviors that violate adolescents’ psychological needs in the development of depressive symptoms of boys and girls in adolescence. At the same time, the use of a six wave longitudinal design in this study helps to disentangle the direction of these effects, and shows the unique importance of boys and girls in shaping the parenting they receive across adolescence. Moreover, these findings give important insight in potential factors for intervention when adolescents experience depressive symptoms. Although adolescents’ depressive symptoms are predicted by mothers’ parenting behaviors, being psychological control, this study shows that both aspects of mothers’ empathic tendencies precede mothers’ psychological control use, thus providing additional information on where to intervene. Additionally, as a community sample was used, the findings provide useful information on factors to address in screening and prevention programs for depressive symptoms in adolescence. Finally, with these findings, this study provides promising bases for future research to further examine the indirect role of both mothers’ and fathers’ empathy in predicting other outcomes of adolescents’ wellbeing, such as anxiety symptoms.

## Footnotes

1 Table with within-wave correlations of all studied variables from age 13 to age 18 for the total sample (*N* = 497) and boys (*N* = 283) and girls (*N* = 214) separately is available from the first author on request
